# Omphalic Bleed

**DOI:** 10.7759/cureus.16066

**Published:** 2021-06-30

**Authors:** Nagarajan Raj Kumar, Muhamed Tajudeen

**Affiliations:** 1 Surgery, Jawaharlal Institute of Postgraduate Medical Education and Research, Puducherry, IND

**Keywords:** omphalic bleed, umbilical varices, sclerosant injection, suture ligation, surgical emergency scenarios

## Abstract

Portosystemic collateralization is usually seen in patients with portal hypertension. Bleeding from the ectopic varices is reportedly rare. We present a case of a 55-year-old gentleman who presented with complaints of bleeding from the umbilicus. On examination, he was tachycardic, hypotensive, and in hypovolemic shock. Bleeding was suspected to be from the umbilical varices. Contrast-enhanced computed tomography of the abdomen with abdominal angiography revealed a cirrhotic liver with partial thrombosis of the portal vein with collaterals in the perigastric, lower esophageal, peripancreatic, splenic and mesenteric, umbilical and paraumbilical collaterals with recanalization of the umbilical vein. The bleeder was identified to be the collateral at the umbilical region from the superior mesenteric vein. The patient was treated with a Doppler-guided injection of a sclerosant into the collateral, thereby achieving successful hemostasis.

## Introduction

One known complication of decompensated chronic liver disease is bleeding from the esophagogastric varices due to portal hypertension. Ectopic varices are defined by large portosystemic venous collaterals occurring anywhere in the gastrointestinal tract, other than the esophagogastric region, which is less common. The sites for ectopic varices are the duodenum, jejunum, ileum, colon, rectum, peritoneum, retroperitoneum, right diaphragm, biliary tree, vaginal vault, ovaries, urinary bladder, and umbilicus region [1]. Bleeding from these ectopic varices constitutes 1%-5% of all variceal bleed in patients with intrahepatic portal hypertension and 20%-30% in patients with extrahepatic portal hypertension [2]. There is scanty literature on the cases of management of omphalic bleed. Most of the literature available is only by case reports and individual experiences. Here, in this case, we report a case of bleeding umbilical varices managed with sclerosant injection successfully.

## Case presentation

**Figure 1:**
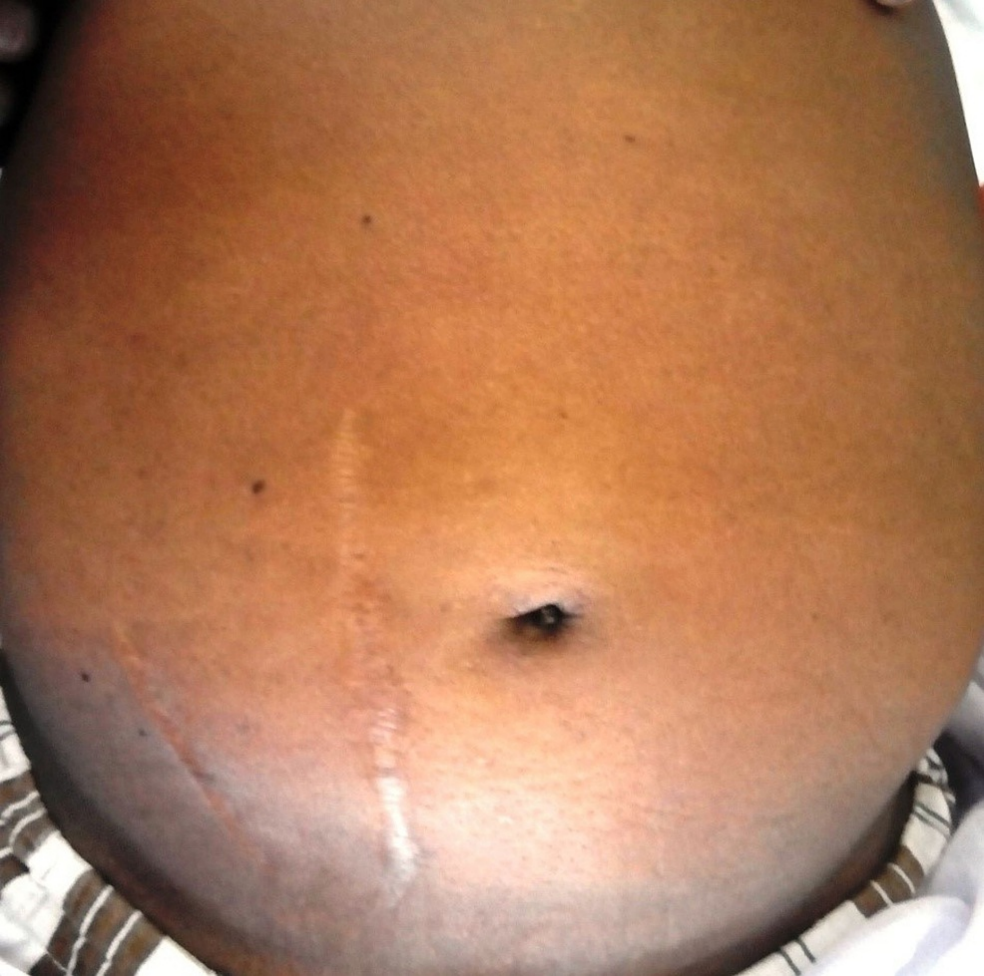
Abdominal examination showing a distended abdomen with no evidence of caput medusae or collaterals with scars in place

We present a case of a 55-year-old gentleman who presented to casualty services with the complaint of bleeding from the umbilicus for the past eight hours, which was spontaneous in onset, and the patient was managed with compression dressing at a local hospital and referred to a higher facility. He had a history of alcohol intake of 90-120 grams a day regularly for 35 years. There were no prior episodes of hospitalization or similar events in the past. On presentation, he was conscious and oriented to time, place, and person. There were no signs of hepatic encephalopathy. He was very pale. He had a feeble pulse at a rate of 130 beats per minute with a blood pressure of 90/50 mmHg. Abdominal examination revealed a soft abdomen with massive ascites, and there was no evidence of active bleeding (Figure 1).

**Figure 2:**
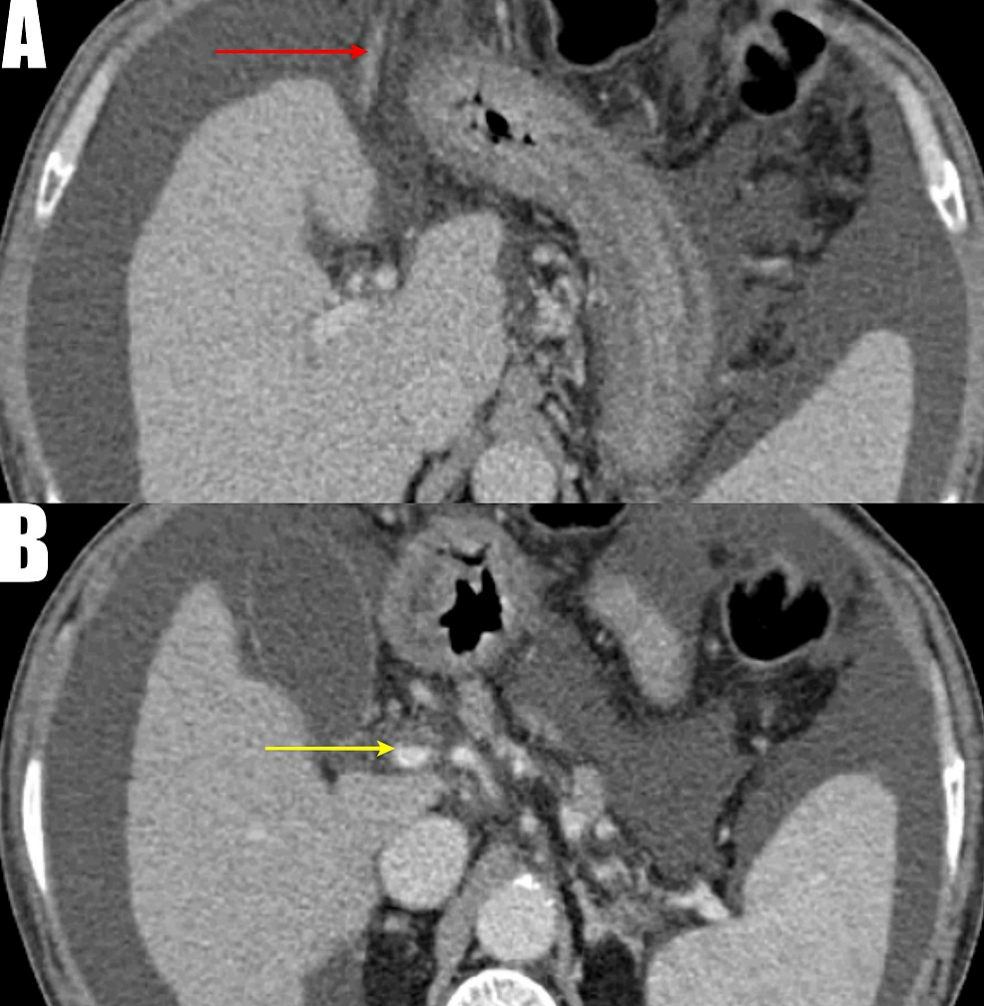
Computed tomography angiogram image A: Cirrhotic liver with massive ascites with a recanalized umbilical vein (red arrow); B: Thrombosis of the main portal vein marked by a yellow arrow

Blood investigations showed a hemoglobin of 4.5 g/dl with normal platelet counts. His international normalized ratio (INR) was 2.8 (normal range=0.8-1.2). The biochemical investigations were normal. His model of end-stage liver disease (MELD) score was 20, and the Child-Turcotte-Pugh (CTP) score was 10. He was resuscitated with crystalloids, packed cells, and fresh frozen plasma. It was when he again bled from umbilical varices, which was initially controlled with suture ligation. Ultrasound abdomen revealed parenchymal liver disease with gross ascites, and diagnostic ascitic tap ruled out intraperitoneal bleed. Upper gastrointestinal endoscopy showed three columns of grade three varices, and banding of the varices was done. Contrast-enhanced computed tomography (CECT) of the abdomen with abdominal angiography showed a cirrhotic liver with thrombosis of the portal vein with collaterals in the umbilical region, perigastric, lower esophageal, peripancreatic, splenic hilar, and mesenteric collaterals with a recanalized umbilical vein noted (Figure 2).

**Figure 3:**
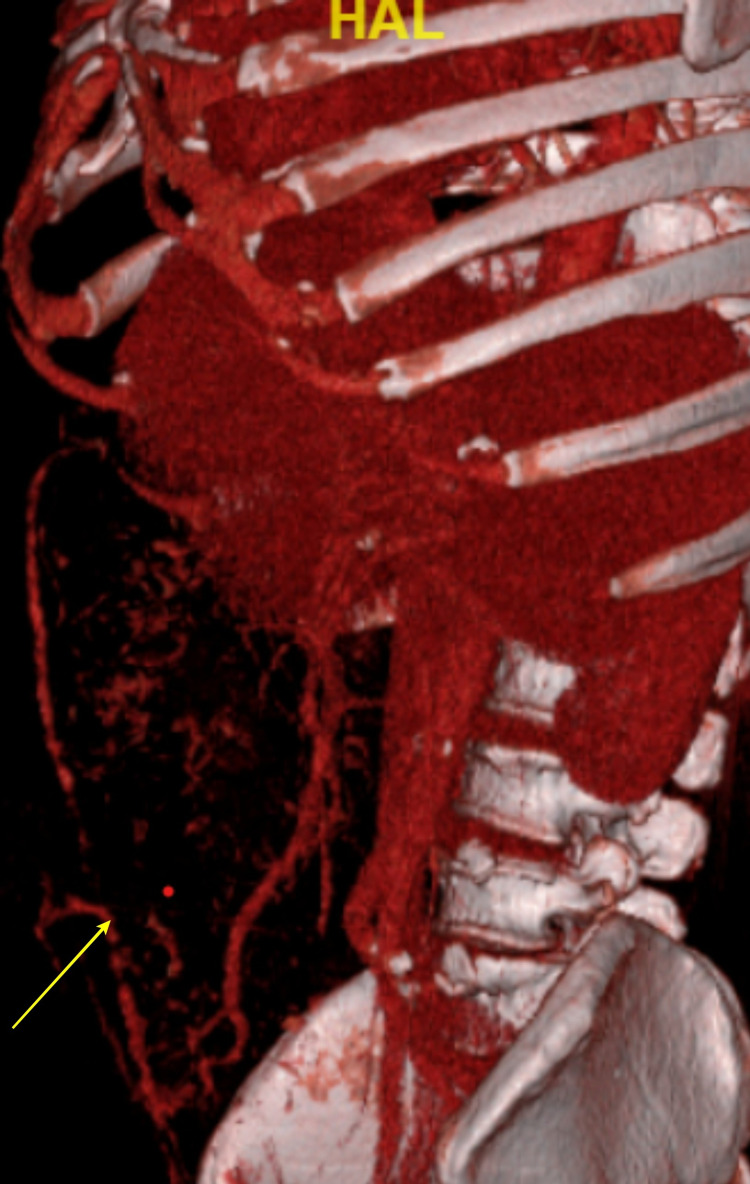
3D reconstruction imaging showing the collateral from the superior mesenteric vein to the umbilical region (yellow arrow)

The bleeder was one of the collaterals communicating to the superior mesenteric vein, opening to the skin surface (Figure 3).

Although suture ligation of the varices was done, with an anticipated risk of rebleed under ultrasound Doppler guidance, sclerotherapy was carried out with 30 milligrams of sodium tetradecyl sulfate, and compression dressing was applied. The patient had an uneventful recovery and discharged with the oral beta-blocker propranolol.

## Discussion

The most common site of bleeding in patients with portal hypertension is gastroesophageal varices. The development of ectopic varices in a patient with portal hypertension can be natural as part of the disease or following iatrogenic opposition of structures with different venous drainage during any abdominal surgery like the creation of stomas. In a study of 169 cases, Norton et al. noted bleeding ectopic varices most commonly occurred in the small bowel [2].

Cruveilhier-Baumgarten syndrome is defined as recanalization of the umbilical vein due to portal hypertension, a known complication of liver cirrhosis [3]. Ectopic paraumbilical varices can develop from a recanalized umbilical vein that originates from the left intrahepatic portal vein traversing through the falciform ligament and drains into superficial epigastric veins over the anterior abdominal wall [4]. The paraumbilical vein collaterals are reported in 10%-29% of patients with portal hypertension [5].

Norton et al. reported a mortality rate of 40% for bleeding ectopic varices at the index presentation [2]. Mortality with ectopic varices is primarily due to the delay in detection of bleeding source, particularly in cases of non-luminal sources like retroperitoneum [6].

Evaluation of the variceal bleed can be done through various investigative modalities. For duodenal and small bowel variceal bleed, upper gastrointestinal endoscopy or double-balloon enteroscopy is indicated. Video capsule endoscopy can be done for small bowel varices. Doppler ultrasonography can be done. Computed tomography angiogram (CTA) can be successful in cases of retroperitoneal and colonic varices.

Currently, there are no randomized controlled trials or guidelines on the management of bleeding umbilical varices. All the available literature is by way of case reports and individual experiences. The management will depend on the site of hemorrhage (intraperitoneal or over the skin), presentation, the expertise of the treating personnel, and underlying causes of portal hypertension. The treatment plan is individualized as per the patient’s status.

The management of bleeding umbilical varices is similar to that of esophagogastric variceal bleeds like clinical assessment, hemodynamic stabilization, antibiotics, and vasoactive agents like octreotide or terlipressin. Other therapeutic options available for controlling bleeding umbilical varices include simple suture ligation of varices and band ligation [7]. Simple suture ligation of umbilical varices over the skin is always only a temporary measure; a definitive procedure should accompany, given high chances for rebleeding. For large varices, band application should be avoided because of incomplete occlusion of the dilated vein. The necrosed part sloughs off, leading to the formation of the larger crater within varix and cause rebleeding. For patients who fall under Child-Pugh class A and extrahepatic portal vein obstruction, the definitive treatment should be shunt surgery. For the patients with Child-Pugh class B and C, cirrhosis can be better managed by procedures like sclerotherapy, embolization, and transjugular intrahepatic portosystemic shunt (TIPS). In addition, performing concurrent embolization for the ectopic varices along with TIPS minimizes the chances of rebleeding [8].

The management of bleeding umbilical varices by sclerotherapy is a safe option in centers with limited resources, is readily available, and can be easily practiced. Treatment modality has to be individualized for each patient depending on the condition and presentation of the patient. In our patient, we used sclerotherapy as a modality for the obliteration of varices. He did not have any recurrence after that for two months.

## Conclusions

Bleeding from umbilical varices due to portal hypertension is rare, and the presentation can be almost lethal at times, as in our case. At present, the management of bleeding umbilical varices is individualized as per the patient’s status and circumstances. Compared with other modalities, sclerotherapy can be safely done with a good outcome. Data on general consensus in such a case is difficult to produce, considering the rarity of the presentation.
